# Editorial: Human-in-the-loop paradigm for assistive robotics

**DOI:** 10.3389/frobt.2025.1718326

**Published:** 2025-11-19

**Authors:** Francesca Cordella, Dario Farina, Loredana Zollo

**Affiliations:** 1 Research Unit of Advanced Robotics and Human-Centred Technologies (CREO Lab), Università Campus Bio-Medico di Roma, Rome, Italy; 2 Department of Bioengineering, Imperial College London, London, United Kingdom

**Keywords:** human-in-the-loop, assistive robotics, human state modeling, robot adaptation, human-robot interaction

## Introduction

The collaboration between humans and robots has become a central theme in the evolution of assistive technologies. In domains such as rehabilitation, prosthetics, and daily living assistance, the paradigm of human-in-the-loop introduces a new perspective: rather than designing robots to act independently, robotic systems are endowed with mechanisms to incorporate human inputs, feedback, and intentions directly into their control and learning processes. This integration facilitates adaptation, personalization, and mutual trust between humans and robotic agents, thereby improving usability and acceptance in real-world scenarios.

A crucial element of this paradigm is the ability to continuously model the human state, encompassing physical, cognitive, and affective dimensions, to capture user intent, fatigue, emotion, and attention in real time. Such online modeling is essential for achieving robots that can dynamically adapt their behaviour to changing human conditions and needs and ensure intuitive, empathetic interaction.

While autonomous robots excel in precision, repeatability, and trajectory execution, humans bring situational awareness, contextual decision-making, and corrective actions that are still difficult for machines to achieve. The human-in-the-loop paradigm combines the strengths of humans and robots to develop systems that are technically effective and designed with the human user at their core. Within assistive robotics, this is particularly critical: the interaction must account for unpredictability, support the user’s individual needs, and guarantee safety, transparency, and trustworthiness.

Despite the significant progress in robotics, outcomes in rehabilitation and assistive domains are still limited. Clinical results from robot-aided therapies often remain comparable to traditional interventions, and prosthetic devices still fall short of replicating the natural behaviour of biological systems. These challenges highlight the need for research on how human feedback, intention recognition, multimodal interaction, and personalized control can be used to improve assistive robotic systems.

This Research Topic on the “Human-in-the-loop paradigm for assistive robotics” brings together contributions that highlight novel methods, experimental studies, and conceptual frameworks addressing these open Research Topic. The Research Topic includes approaches on haptic-augmented teleoperation, explainable trajectory corrections, movement recognition and myoelectric control for muscular dystrophy patients. Together, these works shed light on how human-in-the-loop strategies can improve adaptability, intuitiveness, and effectiveness in assistive robotics.

Across these studies, a common thread is the need to sense and model the user state in real time, enabling robots to adapt continuously and maintain effective and safe interaction. Such capability enables human–robot co-adaptation and paves the way for next-generation assistive systems that can respond not only to physical cues but also to cognitive and emotional feedback.

## Contributed articles

One promising direction in human-in-the-loop robotics lies in enhancing transparency and sensory augmentation during interaction. The study by van den Berg et al. addresses this by integrating force visualization into VR teleoperation environments. By augmenting visual feedback with representations of interaction forces, the system enables operators to better understand and control their actions, particularly in haptic-assisted scenarios. This approach demonstrates how multimodal feedback can increase task performance and user confidence, highlighting the role of perceptual augmentation in the human-in-the-loop framework.

A related challenge is how humans communicate corrections and preferences to robotic systems. Yow et al. introduce a framework where users can provide trajectory corrections through natural language. In particular, the system produces explainable corrections, linking adjustments to textual descriptions of features. This approach advances the transparency and intuitiveness of human-robot communication, bridging the gap between user intentions and robotic adaptation. It highlights how linguistic and symbolic interfaces can expand accessibility, particularly for non-expert users, while maintaining system interpretability.

Understanding human movement is another important aspect to consider for an effective human-in-the-loop interaction. Mignone et al. explore how robots can better recognize human-like motion by comparing passive observation with active interaction. Their findings emphasize that interaction yields richer information than mere observation, improving the accuracy of recognizing and predicting human movements. This insight is critical for applications such as rehabilitation or collaborative assistance, where anticipating and adapting to user behaviour determines system effectiveness.

The human-in-the-loop paradigm also plays a crucial role in personalized assistive technologies for individuals with neuromuscular impairments. Nizamis et al. present a case study on myoelectric control for patients with Duchenne muscular dystrophy. The proposed system allows real-time control of wrist and hand movements, showing that, even in complex and progressive conditions, involving the user in the control loop can help preserve functional autonomy. This study adapts control strategies to the patient’s remaining muscular activity, demonstrating how personalized, user-centred robotics can enhance the quality of life and broaden the impact of assistive technologies.

## Conclusion

This Research Topic shows how the human-in-the-loop paradigm can play an important role in advancing assistive robotics. Across different applications, such as teleoperation, natural language-based interaction, movement recognition, and personalized myoelectric control, the contributions converge on a common theme: leveraging human feedback and involvement to create robotic systems that are adaptive, transparent, and trustworthy. The studies presented highlight how multimodal feedback can improve user understanding and confidence, how explainable and intuitive interaction supports broader acceptance, and how active engagement helps robots recognize human movements more effectively. They also show that personalization, through adapting control strategies to each individual abilities and needs, remains essential for the success of assistive robotics.

Together, these studies position the human-in-the-loop paradigm not merely as a design choice, but as a necessity for advancing assistive robotics toward safe, effective, and user-centred solutions. By fostering adaptive control, transparent communication, and inclusive personalization, human-in-the-loop approaches can bridge the current gap between technological potential and real-world therapeutic outcomes. The Research Topic offers a roadmap for future research at the intersection of robotics, human factors, and healthcare, guiding the development of assistive systems that are not only technically sophisticated, but also deeply aligned with human needs and experiences.

## Future directions

Future developments should therefore focus on building accurate online models of human state and intention, which are fundamental to achieving truly adaptive and responsive assistive robots. Realizing the full potential of the human-in-the-loop paradigm requires moving beyond current integration schemes toward deeper cognitive–affective modeling and mutual learning between human and robot. Future research should focus on adaptive control strategies that account for the user’s physical state, attention, emotion, and preferences in real time. This will allow the development of assistive systems capable of co-evolving with their users, fostering more natural, empathetic, and inclusive interactions. Building on this foundation, the field could benefit from a conceptual framework that links the cognitive, motor, and emotional aspects of human–robot interaction. Such a model would help unify the understanding of how perception, decision-making, and emotion interplay in assistive contexts, paving the way for systems that are not only intelligent and responsive, but also deeply human-centered in both design and ethics.

